# Psychiatric Advance Directives, a Possible Way to Overcome Coercion and Promote Empowerment

**DOI:** 10.3389/fpubh.2014.00037

**Published:** 2014-04-29

**Authors:** Yasser Khazaal, Rita Manghi, Marie Delahaye, Ariella Machado, Louise Penzenstadler, Andrew Molodynski

**Affiliations:** ^1^Geneva University Hospitals, Geneva, Switzerland; ^2^Geneva University, Geneva, Switzerland; ^3^Oxford Health NHS Foundation Trust, Oxford, UK; ^4^Department of Psychiatry, Oxford University, Oxford, UK

**Keywords:** advance directives, cognitive-behavior therapy, coercion, empowerment, recovery

## Abstract

Psychiatric advance directives (ADs) allow an individual to state their preferences for future treatment at times when they may be unable to make considered decisions. There are differences in their form and legal value and the process associated with their use and completion. Several studies have now been completed to assess the impact of ADs on service use and coercion. Their results give a mixed picture but directives nevertheless have the potential to support the empowerment process, minimize experienced coercion, and improve coping strategies. These may in turn reduce the frequency of in-patient service use. Further studies on the different processes of facilitation involved and on different populations are necessary to improve our knowledge and use of these potentially powerful interventions.

## Introduction

Concerns related to compulsory treatment and perceived coercion have been reported in European countries (1, 2). Coercion is a wide ranging and poorly defined term that includes compulsory care in both hospital and the community and also the non-statutory pressures exerted upon patients to accept treatment (3, 4). So-called “voluntary” patients often feel coerced to accept their mental healthcare treatment and this may come from several sources (5). Such coercion has been described as a hierarchy: persuasion, leverage, inducement, threat, and compulsion (6, 7). It appears from large scale studies that such strategies are common (5).

Compulsory treatments have been found to be associated with a number of negative effects (3, 8, 9) such as reduced treatment satisfaction (10) and increased rates of future involuntary treatment (2).

Perceived coercion may be associated with either compulsion or other treatment pressures and may alter the therapeutic alliance (11). Negative treatment experiences could lead to a reduction in future help seeking and may increase avoidance of mental health care and support (12). This would be counter productive and likely to lead to adverse outcomes and reduce autonomy and empowerment over time (4). It is therefore important to develop both measures of perceived coercion and interventions that may reduce it. In this paper, we present psychiatric advance directives (ADs) as a possible intervention to reduce perceived coercion and enhance empowerment (13, 14).

The process of empowerment requires the active participation of the person receiving care in the decision-making process (15). It has been argued that this is the “opposite” of the use of coercion (16).

## Empowerment

Empowerment has been defined as “the ongoing capacity of individuals or groups to act on their own behalf to achieve a greater measure of control over their lives” (15).

Empowerment is a process rather than an outcome (17–20) and is at the heart of the recovery process (21–24).

The main components of empowerment include: access to information and resources, ability to make choices, critical thinking, the assertive expression of needs, self-efficacy and self-esteem, social activism, righteous anger, and optimism-control (25). It has been demonstrated that perceived coercion decreases when individuals have a say in their care decisions (16).

## Psychiatric Advance Directives

Psychiatric ADs are legal documents (albeit with different authority in different jurisdictions) that allow an individual to clearly state their preferences for future treatment at times when they may be unable to make considered decisions. They can thus be considered as a way “to help people retain control over their treatment when incapacitated” (13). ADs thus support the empowerment process in the medium to long-term and have the potential to minimize perceived coercion (16).

As related by a service user, ADs can work as a bridge to self-knowledge (26). The process of creating the plan involves the patient in an active analysis of the past crisis and in developing possible alternative approaches in future.

## Barriers to Implementation

In spite of the potential and real benefits described above mental health service users (27) rarely use directives. Several barriers have been identified that relate to the individual themselves, the care team, and/or the overall system (27).

In most jurisdictions, ADs can be overridden of the risks are considered to be high enough and this remains one of the most important barriers to their use (27). Service users’ confidence in their directives and whether they will be respected are strongly linked to this issue.

A history of recent involuntary treatment (within the last 2 years) has been shown to be associated with a reduced interest in ADs (28). We hypothesize that this may be due in part to a number of factors: fear of legislation, lack of confidence in institutions, and cognitive avoidance of distressing previous crises and their treatment.

Two studies have found however that 50–75% of mental heath service users are attracted by ADs, particularly if support to complete them is available (28, 29).

The contrast between high levels of interest and low levels of uptake (particularly among those recently coerced) demonstrates the need to promote, develop, and assess specific interventions.

## Types of Advance Directives

Directives vary in form, content, and legal authority and there are also significant differences in the way they are drawn up. Typically they fall into one of three broad types: classic psychiatric advance directives (C-AD), facilitated psychiatric advance directives (F-AD), and joint crisis plans (J-CP) (30).

### Classic psychiatric advance directives

Classic psychiatric advance directives are generated by the service user without any specific assistance. The individual may: (1) give informed consent to future treatment, (2) express personal values and treatment preferences, or (3) delegate a proxy for taking decisions in case of future crisis and/or loss of decision-making ability. These directives can be prescriptive (saying what to do), proscriptive (saying what not to do), or both. If chosen with care these can greatly enhance autonomy, but this can be compromised by a lack of support and information. C-AD are sometimes not carried out to their full extent due to lack of clear description of specific crisis-related situation and treatment details. In such cases, the treatment preferences are more likely to be overruled by caregivers.

Due to very low rates of completion overall (27) and difficulties experienced in the process without support (24, 28), alternative types of facilitation have been proposed.

### Facilitated psychiatric advance directives

Facilitated psychiatric advance directives are a form of directive in which a trained facilitator helps the service user document his or her preferences and thus create a final document. A randomized controlled study showed that this form of facilitation significantly increased completion rates (31). The exact means of facilitation varies.

One of the most studied methods of facilitation (31) is a 60–90 min semi structured and manualized discussion with a trained facilitator. It includes the provision of information and a review of past treatment experiences before the development of future treatment preferences.

Advance directives based on cognitive therapy (ADBCT) are another form of directive (32). A trained staff member (ideally the care coordinator) facilitates the process in generally two to eight sessions (33). ADBCT aim to explore “what happened” and develop possible alternatives for future episodes. Such intervention typically requires more than one session, particularly if there is avoidance behavior related to the previous crisis or to perceived coercion (33). The novelty of ADBCT (Table 1) lies in the cognitive-behavioral conceptualization of the process (33). There is a collaborative approach and a “concordance model” (34) founded on joint exploration of the individual’s specific illness model and coping strategies. The whole process is a constructive collaboration between patient and caregiver, in which disagreements and differences of opinion are respected and acknowledged. It also incorporates motivational interviewing and problem solving approaches (35).

**Table 1:** **ADBCT seven steps**.

1.	Information provision regarding advance directives
2.	Exploration of past experiences of mental health crises. Identification of relapse triggers and prodromal signs
3.	Assessment of past experiences of medication and other treatments, both helpful and unhelpful. Evaluation of the cost and benefit of each treatment and in which circumstances a given treatment is acceptable or not
4.	Long-term relapse prevention strategies are discussed including coping strategies which should be used or not if a specific situation arises
5.	The individual drafts the advance directives statement independently but can ask for support if needed. Specific thought is given here to ensuring the directive is readily accessible when needed
6.	During further episodes of crisis, the advance directive will be used if appropriate and its content openly discussed with the originator
7.	After the episode of illness, the directive is rigorously reviewed to ensure it best meets the individual’s particular needs and desires

Facilitated psychiatric ADs may be vulnerable to the influence of the facilitator on the service user so care is needed in this regard during facilitation.

### Joint crisis plans

Joint crisis plans (36, 37) are another form of directive. Here the service user and the care team are involved in a negotiating process with a third-party facilitator. This facilitator might be a mental health worker (from a different part of the service), a family member, a friend, or an advocate. The first appointment helps the patient and his case manager to formulate the plan. During the second meeting, the patient might invite his other caregivers, family members, and psychiatrist in order to finalize the content. The facilitator’s role is to encourage and allow discussion between all parties. If there are continuing differences of opinion these are clearly documented in the plan.

A recent study in the Netherlands compared two types of J-CP, one facilitated through the patient’s advocate and the other by the patient’s clinician (38). The content of the directives was assessed (39) using a “quality of crisis plan checklist.” With the exception of the sub-score related to the medication, the scores were higher in the advocate group than in the clinician group. This may be due to a number of factors (39) such as better patient–advocate relationships, a more holistic approach by advocates, possible patient preferences for non-clinical encounters or to insufficient training in clinicians.

## When is the Best Time to Create a Directive?

There is no consensus on when is the optimal time to create a directive. It has been argued (40) that directives must be drafted in community settings and not during hospitalization. Hospitalization is however where the issues are often most acute and/or difficult.

Others suggest (33) that the end of the admission might be the most appropriate period as the acute crisis has subsided but remains fresh in the person’s memory and there is generally active support from services. Such a collaborative approach at this stage may also serve to reduce treatment dropout following discharge from hospital.

### Transition critical-time

For some, developing a directive will always be difficult whether they are in hospital or in the community. For others (e.g., high users of in-patient care), the critical moment of transition between in-patient and community settings represents a moment of crisis (41) and may be a chance to intervene and develop plans. For these groups, transitional case management (TCM) may be helpful. This is a brief intervention (42) adapted from assertive community treatment (ACT) models (43). It has been suggested (44) that integrating TCM with some forms of ADs may be helpful.

### Legal authority of directives

The usefulness of directives is dependent upon them being used and “respected” by those making decisions during periods of crisis (30). Access may be improved by measures such as coping the directive to the care team and/or lodging them in electronic records systems. The respecting of directives depends on a number of factors: legislation, culture among professionals, and crucially the actual content of the directive.

### Impact on service use and coercion

A Cochrane review *and meta-analysis* (45) on ADs included only two trials. An evaluation of an intensive intervention with a J-CP (36) concluded that participants in the intervention group were less likely to have been re-hospitalized (at a trend level only) either voluntarily or involuntarily at follow-up. No differences were found for the low intensity intervention study based on booklet information (46). The authors hypothesized that “more intensive forms of AD show promise” and concluded: “there are too few data available to make definitive recommendations.” They highlighted the need for good quality randomized controlled trials to assess the effectiveness of ADs in the mental health setting.

These conclusions have to be assessed alongside less positive recent empirical findings (37) not included in the review. The results of this large study did not confirm the previous results (36), possibly due to some “dilution” in the clinicians’ skills or motivation due to involvement of a wider group of clinicians in the study.

In US, a study on F-AD (24) compared a sample of completers to non-completers and found a statistically significant association between F-AD completion and lower rates of coercive interventions at follow-up. The study could not however exclude possible bias from initial differences between completers and non-completers.

The evidence regarding ADs is therefore mixed. A number of controlled studies suggest a reduction in involuntary hospitalization (24, 36) or a trend toward a reduction (36) and other studies do not (37, 46). Positive effects on service use and coercion may therefore be associated with the type or the intensity of the facilitation process used, but further research in this area is required to draw any firm conclusions.

### Possible mechanisms of change

Enthusiasm generated by the emergence of ADs led some to hypothesize an impact upon core recovery processes. It has been asserted that directives empower through the expression of the patient’s own treatment preferences (30, 47, 48). Following any of the processes described above the service user and those around them should be more aware of early signs of relapse and have a clearer picture of acceptable and effective intervention strategies (24, 32, 33, 36, 49). This in turn is likely to lead to the enhancement of insight, self-esteem, and satisfaction with treatment (50). It has been demonstrated that facilitated directives improve the working alliance with clinicians at short-term follow-up (31).

It may also be the case that directives are possible tools for coordinating tasks and enhancing communication and coherence (30). This possible effect on continuity of care could improve an individual’s network and reduce high or “chaotic” in-patient and emergency rooms service use (36, 46, 48, 51). Further enquiry is required however.

### Future directions

Further studies (45) are required to examine the more intensive forms of intervention described above.

The most frequently studied interventions (F-AD or J-CP) are generally very short-term and there may be a need in complex situations and difficult situations for more sessions to develop more complex and considered plans. More intensive interventions such as ADBCT (32, 33) may therefore be valuable alternatives. Further studies are needed to substantiate this and better understand the processes involved.

High users of services may particularly benefit from a facilitated approach as their care is often crisis driven and chaotic, but existing research does not focus on this particular group. Only 10% of the patients included in the larger study on J-CP (37) had three or more hospital admissions in the 2-years before study inclusion. The participants of the intervention group of the larger study on C-AD had a mean of 0.6 ± 0.3 in-patient admissions in the year before inclusion. Further studies on high service users (in-patient services as well as emergency rooms) are clearly needed.

## Conclusion

There are important differences in the processes involved in completing ADs, in their content, and in their legal authority between jurisdictions. There are also clear variations in individual and group practice that may serve to limit their use. These differences are likely to reduce the potential of ADs to influence such outcomes as service use, the empowerment process, and recovery. They may also lead to individuals feeling a greater sense of coercion. Some encouraging research findings however suggest that ADs may have an impact upon one or more of these outcomes. Further studies are urgently needed on the different forms of directive and the processes of facilitation involved to allow us to develop and target their use more clearly and effectively. Research on populations who may benefit in terms of their diagnosis and patterns of service use are also required to ensure that those who may benefit most are allowed the opportunity. The conclusions of the present review need to be considered in the light of the fact that is a narrative review. Systematic reviews and meta-analysis of the emerging evidence (37) in this field and ongoing large scale studies (48, 52) should significantly improve our understanding over the coming years.

## Conflict of Interest Statement

The authors declare that the research was conducted in the absence of any commercial or financial relationships that could be construed as a potential conflict of interest.

## References

[1] Raboch J Kalisova L Nawka A Kitzlerova E Onchev G Karastergiou A Use of coercive measures during involuntary hospitalization: findings from ten European countries. Psychiatr Serv (2010) 61(10):1012–7 10.1176/appi.ps.61.10.1012 20889640

[2] van der Post LF Peen J Dekker JJ A prediction model for the incidence of civil detention for crisis patients with psychiatric illnesses; the Amsterdam study of acute psychiatry VII. Soc Psychiatry Psychiatr Epidemiol (2014) 49(2):283–90 10.1007/s00127-013-0742-7 23863912

[3] Bonsack C Borgeat F Perceived coercion and need for hospitalization related to psychiatric admission. Int J Law Psychiatry (2005) 28(4):342–7 10.1016/j.ijlp.2005.03.008 15936078

[4] Khazaal Y Bonsack C Borgeat F [Coercion in psychiatry: current knowledge and perspectives]. Rev Med Suisse (2005) 1(7):515–8 15790020

[5] Burns T Yeeles K Molodynski A Nightingale H Vazquez-Montes M Sheehan K Pressures to adhere to treatment (‘leverage’) in English mental healthcare. Br J Psychiatry (2011) 199(2):145–50 10.1192/bjp.bp.110.086827 21804149

[6] Molodynski A Rugkasa J Burns T Coercion and compulsion in community mental health care. Br Med Bull (2010) 95:105–19 10.1093/bmb/ldq015 20501486

[7] Szmukler G Appelbaum P Treatment pressures, leverage, coercion, and compulsion in mental health care. J Ment Health (2008) 17:233–44 10.1093/bmb/ldq015 20501486

[8] Rose DS Wykes TH Bindman JP Fleischmann PS Information, consent and perceived coercion: patients’ perspectives on electroconvulsive therapy. Br J Psychiatry (2005) 186:54–9 10.1192/bjp.186.1.54 15630124

[9] Rusch N Muller M Lay B Corrigan PW Zahn R Schonenberger T Emotional reactions to involuntary psychiatric hospitalization and stigma-related stress among people with mental illness. Eur Arch Psychiatry Clin Neurosci (2014) 264(1):35–43 10.1007/s00406-013-0412-5 23689838

[10] Priebe S Katsakou C Amos T Leese M Morriss R Rose D Patients’ views and readmissions 1 year after involuntary hospitalisation. Br J Psychiatry (2009) 194(1):49–54 10.1192/bjp.bp.108.052266 19118325

[11] Kinderman P Tai S Psychological models of mental disorder, human rights, and compulsory mental health care in the community. Int J Law Psychiatry (2008) 31(6):479–86 10.1016/j.ijlp.2008.09.005 18954904

[12] Swartz MS Swanson JW Hannon MJ Does fear of coercion keep people away from mental health treatment? Evidence from a survey of persons with schizophrenia and mental health professionals. Behav Sci Law (2003) 21(4):459–72 10.1002/bsl.539 12898502

[13] Henderson C Flood C Leese M Thornicroft G Sutherby K Szmukler G Views of service users and providers on joint crisis plans: single blind randomized controlled trial. Soc Psychiatry Psychiatr Epidemiol (2009) 44(5):369–76 10.1007/s00127-008-0442-x 18836881

[14] Wilder CM Elbogen EB Moser LL Swanson JW Swartz MS Medication preferences and adherence among individuals with severe mental illness and psychiatric advance directives. Psychiatr Serv (2010) 61(4):380–5 10.1176/appi.ps.61.4.380 20360277 PMC3676902

[15] Linhorst DM Hamilton G Young E Eckert A Opportunities and barriers to empowering people with severe mental illness through participation in treatment planning. Soc Work (2002) 47(4):425–34 10.1093/sw/47.4.425 12450013

[16] La Fond JQ Srebnik D The impact of mental health advance directives on patient perceptions of coercion in civil commitment and treatment decisions. Int J Law Psychiatry (2002) 25(6):537–55 10.1016/S0160-2527(02)00182-6 12414021

[17] Kieffer CH Citizen empowerment: a developmental perspective. Prev Hum Serv (1983) 3(2–3):9–36 10.1300/J293v03n02_03 10266759

[18] McLean A Empowerment and the psychiatric consumer/ex-patient movement in the United States: contradictions, crisis and change. Soc Sci Med (1995) 40(8):1053–71 10.1016/0277-9536(94)00179-W 7597459

[19] Rappaport J Empowerment meets narrative: listening to stories and creating settings. Am J Community Psychol (1995) 23(5):795–807 10.1007/BF02506992 8851350

[20] Salzer MS Consumer empowerment in mental health organizations: concept, benefits, and impediments. Adm Policy Ment Health (1997) 24(5):425–34 10.1007/BF02042724 9239946

[21] Buckles B Brewer E Kerecman J Mildred L Ellis A Ryan J Beyond stigma and discrimination: challenges for social work practice in psychiatric rehabilitation and recovery. J Soc Work Disabil Rehabil (2008) 7(3–4):232–83 10.1080/15367100802487499 19064431

[22] Corrigan PW Empowerment and serious mental illness: treatment partnerships and community opportunities. Psychiatr Q (2002) 73(3):217–28 10.1023/A:1016040805432 12143083

[23] Stromwall LK Hurdle D Psychiatric rehabilitation: an empowerment-based approach to mental health services. Health Soc Work (2003) 28(3):206–13 10.1093/hsw/28.3.206 12971284

[24] Swanson JW Swartz MS Elbogen EB Van Dorn RA Wagner HR Moser LA Psychiatric advance directives and reduction of coercive crisis interventions. J Ment Health (2008) 17(3):255–67 10.1080/09638230802052195 20221301 PMC2835342

[25] Rogers ES Chamberlin J Ellison ML Crean T A consumer-constructed scale to measure empowerment among users of mental health services. Psychiatr Serv (1997) 48(8):1042–7 9255837 10.1176/ps.48.8.1042

[26] Gross R (2011). Available from: http://raoulgross.info/wp-content/uploads/2011/02/L_auteur.pdf

[27] Shields LS Pathare S van der Ham AJ Bunders J A review of barriers to using psychiatric advance directives in clinical practice. Adm Policy Ment Health (2013). 10.1007/s10488-013-0523-3 24248818

[28] Srebnik DS Russo J Sage J Peto T Zick E Interest in psychiatric advance directives among high users of crisis services and hospitalization. Psychiatr Serv (2003) 54(7):981–6 10.1176/appi.ps.54.7.981 12851434

[29] Backlar P A choice of one’s own research advance directives: anticipatory planning for research subjects with fluctuating or prospective decision making impairments. Account Res (1999) 7(2–4):117–28 10.1080/08989629908573946 11658170

[30] Nicaise P Lorant V Dubois V Psychiatric advance directives as a complex and multistage intervention: a realist systematic review. Health Soc Care Community (2013) 21(1):1–14 10.1111/j.1365-2524.2012.01062.x 22452515

[31] Swanson JW Swartz MS Elbogen EB Van Dorn RA Ferron J Wagner HR Facilitated psychiatric advance directives: a randomized trial of an intervention to foster advance treatment planning among persons with severe mental illness. Am J Psychiatry (2006) 163(11):1943–51 10.1176/appi.ajp.163.11.1943 17074946 PMC3747558

[32] Khazaal Y Chatton A Pasandin N Zullino D Preisig M Advance directives based on cognitive therapy: a way to overcome coercion related problems. Patient Educ Couns (2009) 74(1):35–8 10.1016/j.pec.2008.08.006 18829211

[33] Khazaal Y Richard C Matthieu-Darekar S Quement B Kramer U Preisig M Advance directives in bipolar disorder, a cognitive behavioural conceptualization. Int J Law Psychiatry (2008) 31(1):1–8 10.1016/j.ijlp.2007.11.001 18162188

[34] Scott J Tacchi MJ A pilot study of concordance therapy for individuals with bipolar disorders who are non-adherent with lithium prophylaxis. Bipolar Disord (2002) 4(6):386–92 10.1034/j.1399-5618.2002.02242.x 12519098

[35] Miller WR Motivational interviewing: research, practice, and puzzles. Addict Behav (1996) 21(6):835–42 10.1016/0306-4603(96)00044-5 8904947

[36] Henderson C Flood C Leese M Thornicroft G Sutherby K Szmukler G Effect of joint crisis plans on use of compulsory treatment in psychiatry: single blind randomised controlled trial. BMJ (2004) 329(7458):136 10.1136/bmj.38155.585046.63 15240438 PMC478218

[37] Thornicroft G Farrelly S Szmukler G Birchwood M Waheed W Flach C Clinical outcomes of joint crisis plans to reduce compulsory treatment for people with psychosis: a randomised controlled trial. Lancet (2013) 381(9878):1634–41 10.1016/S0140-6736(13)60105-1 23537606

[38] Ruchlewska A Mulder CL Smulders R Roosenschoon BJ Koopmans G Wierdsma A The effects of crisis plans for patients with psychotic and bipolar disorders: a randomised controlled trial. BMC Psychiatry (2009) 9:41 10.1186/1471-244X-9-41 19589145 PMC2716324

[39] Ruchlewska A Mulder CL Van der Waal R Kamperman A Van der Gaag M Crisis plans facilitated by patient advocates are better than those drawn up by clinicians: results from an RCT. Adm Policy Ment Health (2014) 41(2):220–7 10.1007/s10488-012-0454-4 23238910

[40] Thornicroft G Farrelly S Birchwood M Marshall M Szmukler G Waheed W CRIMSON [crisis plan impact: subjective and objective coercion and engagement] protocol: a randomised controlled trial of joint crisis plans to reduce compulsory treatment of people with psychosis. Trials (2010) 11:102 10.1186/1745-6215-11-102 21054847 PMC2992058

[41] Qin P Nordentoft M Suicide risk in relation to psychiatric hospitalization: evidence based on longitudinal registers. Arch Gen Psychiatry (2005) 62(4):427–32 10.1001/archpsyc.62.4.427 15809410

[42] Dixon L Goldberg R Iannone V Lucksted A Brown C Kreyenbuhl J Use of a critical time intervention to promote continuity of care after psychiatric inpatient hospitalization. Psychiatr Serv (2009) 60(4):451–8 10.1176/appi.ps.60.4.451 19339319

[43] Stein LI Test MA Alternative to mental hospital treatment. I. Conceptual model, treatment program, and clinical evaluation. Arch Gen Psychiatry (1980) 37(4):392–7 10.1001/archpsyc.1980.01780170034003 7362425

[44] Manghi R Rathelot T Khazaal Y Advance directives and addictions, new clinical perspectives? Schweiz Arch Neurol Psychiatr (2012) 163(3):92–7 Available from: http://www.sanp.ch/docs/2012/2012-03/2012-03-013.PDF

[45] Campbell LA Kisely SR Advance treatment directives for people with severe mental illness. Cochrane Database Syst Rev (2009) 1:CD005963 10.1002/14651858.CD005963.pub2 19160260 PMC4161493

[46] Papageorgiou A King M Janmohamed A Davidson O Dawson J Advance directives for patients compulsorily admitted to hospital with serious mental illness. Randomised controlled trial. Br J Psychiatry (2002) 181:513–9 10.1192/bjp.181.6.513 12456522

[47] Elbogen EB Swanson JW Appelbaum PS Swartz MS Ferron J Van Dorn RA Competence to complete psychiatric advance directives: effects of facilitated decision making. Law Hum Behav (2007) 31(3):275–89 10.1007/s10979-006-9064-6 17294136 PMC3816515

[48] Moran P Borschmann R Flach C Barrett B Byford S Hogg J The effectiveness of joint crisis plans for people with borderline personality disorder: protocol for an exploratory randomised controlled trial. Trials (2010) 11:18 10.1186/1745-6215-11-18 20178572 PMC2837648

[49] Srebnik DS La Fond JQ Advance directives for mental health treatment. Psychiatr Serv (1999) 50(7):919–25 10402612 10.1176/ps.50.7.919

[50] Trivedi P Wykes T From passive subjects to equal partners: qualitative review of user involvement in research. Br J Psychiatry (2002) 181:468–72 10.1192/bjp.181.6.468 12456515

[51] Atkinson JM Garner HC Gilmour WH Models of advance directives in mental health care: stakeholder views. Soc Psychiatry Psychiatr Epidemiol (2004) 39(8):673–80 10.1007/s00127-004-0788-7 15300379

[52] Lay B Salize HJ Dressing H Rusch N Schonenberger T Buhlmann M Preventing compulsory admission to psychiatric inpatient care through psycho-education and crisis focused monitoring. BMC psychiatry (2012) 12:136 10.1186/1471-244X-12-136 22946957 PMC3532124

